# Metformin for cancer and aging prevention: is it a time to make the long story short?

**DOI:** 10.18632/oncotarget.6347

**Published:** 2015-11-18

**Authors:** Vladimir N. Anisimov

**Affiliations:** ^1^ Department of Carcinogenesis and Oncogerontology, N.N.Petrov Research Institute of Oncology, St.Petersburg, Russian Federation

**Keywords:** metformin, antidiabetic biguanides, cancer prevention, aging, rodents

## Abstract

During the last decade, the burst of interest is observed to antidiabetic biguanide metformin as candidate drug for cancer chemoprevention. The analysis of the available data have shown that the efficacy of cancer preventive effect of metformin (MF) and another biguanides, buformin (BF) and phenformin (PF), has been studied in relation to total tumor incidence and to 17 target organs, in 21 various strains of mice, 4 strains of rats and 1 strain of hamsters (inbred, outbred, transgenic, mutant), spontaneous (non- exposed to any carcinogenic agent) or induced by 16 chemical carcinogens of different classes (polycycIic aromatic hydrocarbons, nitroso compounds, estrogen, etc.), direct or indirect (need metabolic transformation into proximal carcinogen), by total body X-rays and γ- irradiation, viruses, genetic modifications or special high fat diet, using one stage and two-stage protocols of carcinogenesis, 5 routes of the administration of antidiabetic biguanides (oral gavage, intraperitoneal or subcutaneous injections, with drinking water or with diet) in a wide ranks of doses and treatment regimens. In the majority of cases (86%) the treatment with biguanides leads to inhibition of carcinogenesis. In 14% of the cases inhibitory effect of the drugs was not observed. Very important that there was no any case of stimulation of carcinogenesis by antidiabetic biguanides. It was conclude that there is sufficient experimental evidence of anti-carcinogenic effect of antidiabetic biguanides.

## INTRODUCTION

Cancer prevention is one of main goals of fundamental and clinical oncology. As it was stressed in the preamble to the IARC Handbooks of Cancer Prevention, preventive strategies embrace chemical, immunological, dietary and behavioral interventions that may retard, block or reverse carcinogenic processes or reduce underlying risk factors.[1]

In fourteen volumes of this series of handbooks were collected and critically evaluated available data on cancer preventing effect of non-steroidal anti-inflammatory drugs, carotenoids, vitamin A, retinoids, indoles and isothiocyanates, some fruits and vegetables, tobacco and body weight control programs, breast and cervix cancer screening programs (www.iarc.fr). During the last decade there is a burst of interest to cancer prevention potential of antidiabetic biguanide metformin. More than 120 million prescriptions of MF are written yearly for treatment of type 2 diabetes mellitus and this may have already saved more people from cancer death than any drug in history.[2] Recently it was announced an ambition project of clinical trial called TAME (Targeting Aging with Metformin) proposed by Nir Barzilai and colleagues (Albert Einstein College of Medicine in New York).[3, 4]. They are going to give MF during 5-7 years to 3,000 people aged 70-80 years who already has one or two of three age-associated diseases (heart disease, cancer, cognitive decline). Authors are expected delay these pathologies and death. Persons with type 2 diabetics (T2D) cannot be enrolled due to they were already treated with MF. This project based on the data on the increase of life span of mice and rats given MF and on results of clinical observation on 15% increase in survival of T2D patients primarily treated with MF as compared with healthy people without diabetes. [5] These findings and planning of the TAME clinical trial rise the question on the safety of long-term administration of MF in non-diabetic people.

In present article, we evaluated available results of preclinical studies on anti-carcinogenic and geroprotector effects of metformin (N,N-dimethylbiguanide, MF) and other antidiabetic biguanides phenformin (1-phenylethylbiguanide, PF) and buformin (1-butylbiguanide hydrochloride, BF) and perspectives of its wide introduction in clinical practice. We focused mainly on end-point results of studies, doses and route of administration of biguanides to get answers to two critical questions: (1) are biguanides effective in cancer prevention in non-diabetic organism? and (2) are they safe in long-term treatment? Mechanisms of geroprotective, anti-carcinogenic and antitumor effects of biguanides are intensively studied at present. There are a lot of comprehensive reviews on this topic, [6-15] which could be recommend to readers.

### Antidianetic biguanides: Milestones in the drugs history

In the early 1900s, guanidine was identified as active compound of botanic medicine *Galega officinalis* (French Lilac) commonly used in midevial Europe for the treatment of polyurea in diabetic patients.[15] This was followed by development of antidiabetic biguanides in the 1920s, however due to the discovery of insulin in 1921 only thirty years late the first biguanides (phenformin, buformin and metformin) were synthesized. The drugs were approved in the middle of last century in the USA an Europe for the treatment of T2D.

In the 1971, Dilman [16] proposed that antidiabetic biguanides may be promising as a potent anti-aging and anti-cancer drugs. In the middle of 1970s Dilman at the N.N. Petrov Research Institute of Oncology (St.Petersburg, Russia) initiated the series of experiments in mice and rats to test this hypothesis. In 1974, it was shown in the first time that PF inhibits 7,12-dimethylbenz(a)anthracene (DMBA)-induced mammary carcinogenesis in female rats.[17] Five years later, the first article on the inhibitory effect of PF on spontaneous mammary carcinogenesis and life span extension in cancer-prone female C3H/Sn mice has been published. [18, 19] At the same period it have been reported on inhibitory effect of both PF and BF on spontaneous carcinogenesis in female rats, [20, 21] and on carcinogenesis induced by DMBA or N-nitrosomethylurea (NMU) in mammary gland [22-24], by 1,2-dimethylhydrazine (DMH)–in colon [25, 26], by N-nitrosoethylurea (NEU) and N-nitrosomethylurea (NMU) in kidney and nervous system [27, 28], by 20-methylcholanthrene–in soft tissues, [29] by X-rays - in lymphoid tissue [30] In 1987, we summarized the results of experimental studies on anti-carcinogenic effects of antidiabetic biguanides in special section of our book “Carcinogenesis and Aging”, Boca Raton, FL: CRC Press, 1987, vol.2. [31]

Dilman [32] proposed the idea of metabolic rehabilitation for cancer patients giving them biguanides and maintaining on calorie restricted diet. Treatment with PF decreased incidence of metastases and increased 5-year survival in breast, stomach and colon cancers patients [33, 34] In the late 1970s due to high incidence of adverse effects (severe lactic acidosis) phenformin was forbidden for clinical use and metformin possessed in much less adverse effects started toward nowadays.[15, 34]

After the wave of publications on anti-carcinogenic effect of PF and BF in 1974-1985 with absolute peak in 1980-1982, during next 20 years only one report appeared on inhibitory effect of MF on chemical carcinogenesis of pancreas in hamsters.[35] It seems that interest to this effect was lost, and these very impressive results have been forgotten. In 2003 we have published results of our early studies on anti-carcinogenic and geroprotective effects of antidiabetic biguanides recalculated at Max-Plank Institute for Demographic Research at Rostock, Germany [36] and started new series of experiments using metformin.

In 2005 we firstly have found that treatment with MF inhibits mammary carcinogenesis and increases the life span of female HER-2/neu transgenic mice. [37] At the same year, Evans et al. [38] and then Bowker et al. [39] reported that treatment with metformin decreased the breast cancer risk. These studies induced the second huge wave, like tsunami - an exponential growth of number of publications on MF and cancer. A search in the PubMed on key words “metformin and cancer” shows 1 publication in 1995; 23 articles in 2000, 88 - in 2005, 175 - in 2010, 281 - in 2012, 350 - in 2013, 408 - in 2014. From 1^st^ January to 1^st^ October, 2015 - 330 articles on the topic appeared in PubMed! There are a near hundred excellent reviews and meta-analyzes of epidemiological data on MF effect on cancer risk in type 2 diabetes patients. We refer readers to some of them.[40-44]

During 2005-2010 nineteen original research articles with results of studies on anti-carcinogenic effect of MF in rodent models have been published, and in 2011-2015 – 27 papers. The available data on the results of *in vivo* studies on effects of biguanides involved more than 20 experimental models of carcinogenesis are summarized in the Table 1. They included models of spontaneous carcinogenesis (in rat and mice), chemical carcinogenesis induced by 16 different chemical carcinogens and tumor promoters, viruses, dietetic modifications, ionizing irradiation, transgenic and knockout mice. Antidiabetic biguanides were given with diet, drinking water, orally (gavage), intraperitoneally or subcutaneously. To the date, we found in total 65 publications reported results of original studies. Experiments have been performed on 21 strains of mice (8 inbred, 3 outbred,10 genetically modified mouse strains), 4 rat strains and 1 - hamsters. Effect of antidiabetic biguanides have been studied in 17 target organs/tissues. The majority was focused on mammary gland (21 experiments) and colon tumors (11studies), uterus (5) and liver (7) that reflects the importance of these localizations for clinical practice. Effect of biguanides on total tumor incidence was evaluated in 8 studies. Positive results only (inhibitory effect) induced by treatment with biguanides have been observed in 13 localizations (76%). In colon cancer models, 8 of 11 studies (72.7%) revealed of positive effect of biguanides, whereas in mammary tumor the inhibitory effect of these drugs was shown 15 out 21 experiments (71.4%) and 6 studies (28.6%) failed to show inhibitory effect of the drugs. It worthy to note that there were no cases observed of stimulation of any type of carcinogenesis with antidiabetic biguanides. Only in one study the obese and diabetic mice were used.[87] In all studies on effect of biguanides on induced carcinogenesis, young adult rodents were commonly used, and only in one article the results of the treatment with MF started at the young, adult and old age have been published.[91] Also, only one study was focused on tumorigenesis in mice neonatally exposed to MF.[90] Comprehensive analysis of the results of experimental studies on anti-carcinogenic effect of antidiabetic biguanides has been done recently. [92-104]

**Table 1:** Effects of antidiabetic biguanides on spontaneous and induced carcinogenesis in rodents

Target organ	Species, strain	Sex	Carcinogenic agent^1^	Drug^2^	Dose	Route^3^	Effect^4^	References
Mammary gland	C3H/Sn mice	F	MMTV	PF	2 mg/mouse	oral	↓	[18, 19]
	FVB/N mice	F	HER-2/neu	MF	100 mg/kg	d.w.	↓	[37,45]
	FVB/N mice	F	HER-2/neu	MF	250 mg/kg	i.p.	↓	[46]
	FVB/N mice	F	MMTV-neu;p53+/−	MF	1500 mg/kg	diet	=	[47]
	FVB/N-Tg mice	F	MMTV-PyVT	PF	1.65 mg/ml	d.w.	↓	[48]
	FVB/N-Tg mice	F	MMTV-PyVT	MF	2 mg/ml	d.w.	↓	[48]
	SHR mice	F	Spontaneous	MF	100 mg/kg	d.w.	=	[49]
	LIO rats	F	Spontaneous	PF	2 mg/rat	oral	↓	[21, 36]
	LIO rats	F	DMBA	PF	5 mg/rat	oral	↓	[17, 22]
	LIO rats	F	DMBA	BF	5 mg/rat	oral	↓	[24]
	LIO rats	F	NMU	PF	1 mg/rat	oral	↓	[23]
	SD rats	F	NMU	MF	5 mg/kg 50 mg/kg	oral oral	= ↓	[50]
	SD rats	F	NMU	MF	110-220 mg/kg	diet	↓	[51]
	SD rats	F	NMU	MF	5-50 mg/kg	diet	=	[47]
	SD rats	F	NMU	MF	9.3 mmol/kg	diet	=	[52]
	SD rats	F	NMU	PF	5 mmol/kg	diet	=	[52]
	SD rats	F	NMU	BF	7.6 mmol/kg	diet	↓	[52]
	Wistar rats	F	NMU+OV X	MF	2 mg/mL	d.w.		[53]
	LIO rats	F	X-rays	PF	5 mg/rat	oral	↓	[30]
Pituitary gland	LIO rats	F	Spontaneous	BF	5 mg/rat	oral	↓	[20]
	LIO rats	F	X-rays	PF	5 mg/rat	oral	↓	[30]
Thyroid gland	LIO rats	F	Spontaneous	BF	5 mg/rat	oral	↓	[20]
	LIO rats	F	Spontaneous	PF	2 mg/rat	oral	↓	[21]
Skin	SHR mice	F	BP	MF	200 mg/L	d.w.	↓	[54]
	SHR mice	F	BP	MF	200 mg/L	d.w.	↓	[55]
	FVB/N mice	F	DMBA+TPA	MF	2-250 mg/kg	d.w.	↓	[56]
Soft tissues	Outbred mice	M	MCA	PF	5 mg/kg	oral	↓	[29]
	SHR mice	F	BP	MF	200 mg/L	d.w.	↓	[57]
Uterus	Balb/c mice	F	E2, tamoxifen	MF	50 mg/kg	diet	↓	[58]
	129/Sv mice	F	Spontaneous	MF	600 mg/L	d.w.	↓	[59]
	F344 rats	F	Spontaneous	PF	400-800 ppm	diet	↓	[60]
	LIO rats	F	Spontaneous	PF	2 mg/rat	oral	↓	[21, 36]
	LIO rats	F	X-rays	PF	5 mg/rat	oral	↓	[30, 36]
Cervix utery	SHR mice	F	BP	MF	200 mg/L	d.w.	↓	[61]
Lung	A/J mice	M&F	NNK	MF	250 mg/kg	i.p.	↓	[62]
	LID mice	M&F	NNK	MF	250 mg/kg	d.w.	↓	[63]
	129/Sv mice	M	Urethan	MF	200 mg/L	d.w.	↓	[64]
	Swiss H mice	M&F	Tobacco smoking	MF	800 pm	diet	↓	[65]
Oral mucosa	C57BL/6 mice	F	4-NQO	MF	50 mg/kg	i.p.	↓	[66]
Pancreas	Hamsters	M	NBOPA	MF	320 mg/kg	oral	↓	[35]
	p48Cre/+. LSL-ras G12D mice	M&F	Spontaneous	MF	1000-2000 ppm	diet	↓	[67]
	KPC mice	M&F	Spontaneous	MF	1 mg/ml	d.w.	↓	[68]
	LSA+L-Kras; trp53 mice	M&F	Adenovirus	MF	125 mg/kg	i.p.	↓	[69]
Pancreatic islets	CD1 mice	M	HCFD	MF	50-250 mg/kg	diet	↓	[70]
Liver	C57BL/6 mice	M	NDEA	MF	250 mg/kg	diet	↓	[71]
	C57BL/6 mice	M	Spontaneous	MF	10 mg/kg	diet	↓	[72]
	C57BL/6 (HBxTg) mice	M	HBx transgenic	MF	250 vg/kg	oral	=	[73]
	C57BL/6 mice	M	High fat diet	MF	250 mg/kg	d.w.	↓	[74]
	db/db mice	F	NDEA	MF	300 mg/kg	d.w.	↓	[75]
	Wistar rats	M	NDEA	MF	125 mg/kg	oral	↓	[76]
	Wistar rats	M	NDEA	MF	250 mg/kg	oral	↓	[77]
Small intestines	ApcMin/+ mice	M&F	Spontaneous	MF	250 mg/kg	diet	↓	[78]
	PTEN+/− mice	M&F	Spontaneous	MF	300 mg/kg	d.w.	=	[79]
	PTEN+/− mice	M&F	Spontaneous	PF	300 mg/kg	d.w.	=	[79]
Colon	BALB/c mice	M&F	AOM	MF	250 mg/kg	diet	↓	[80]
	BALB/c mice	M&F	AOM	MF	250 mg/kg	d.w.	↓	[81]
	BALB/c mice	M&F	AOM	MF	250 mg/kg	i.p.	↓	[81]
	Swiss mice	M	DMH	MF	100-200 mg/kg	diet	↓	[82]
	IСR mice	M	DMH + DSS	MF	240 mg/kg	diet	↓	[83]
	LIO rats	F	DMH	PF	5 mg/rat	oral	↓	[25, 26]
	F344 rats	M	AOM	MF	15 mg/kg	d.w.	↓	[80]
	F344 rats	M&F	AOM	MF	500-1000 ppm	diet	=	[84]
	F344 rats	M&F	AOM	MF	1000 ppm	diet	=	[85]
	F344 rats	M&F	AOM	MF	1000 ppm	diet	=	[86]
	SD rats	M	DMH+STZ	MF	150 mg/kg	oral	↓	[87]
	Wistar	M	DMH	MF	100-300 mg/kg	oral	↓	[88]
Kidney	Swiss H mice	M&F	Tobacco smoking	MF	800 ppm	diet	↓	[65]
	LIO rats	M&F	NEU, t.pl.	BF	5 mg/rat	oral	↓	[28]
Lymphoid tissue	B6C3F1 mice	M&F	Spontaneous	PF	300-625 mg/kg	diet	↓	[60]
	PTEN+/− mice	M&F	Spontaneous	MF	300 mg/kg	d.w.	=	[79]
	PTEN+/− mice	M&F	Spontaneous	PF	300 mg/kg	d.w.	↓	[79]
	C57BL/6-Ly5.2	MF	γ-irradiation	MF	250 mg/kg	oral	↓	[89]
	LIO rats	F	X-rays	PF	5 mg/rat	oral	↓	[30]
Nervous suystem	LIO rats	M&F	NMU, tr.pl.	BF	5 mg/rat	oral	↓	[27]
	LIO rats	M&F	NEU, tr.pl.	BF	5 mg/rat	oral	↓	[28]
Total tumors	B6C3F1mice	M&F	Spontaneous	PF	400-800 ppm	diet	=	[60]
	SHR mice	F	Spontaneous	MF	600 mg/L	d.w.	=	[49]
	129/Sv mice	M&F	Spontaneous	MF	600 mg/L	d.w.	↓	[59]
	129/Sv mice	M	Spontaneous	MF	100 mg/kg	s.c.	↓	[90]
	129/Sv mice	F	Spontaneous	MF	100 mg/kg	s.c.	=	[90]
	B6C3F1 mice	M	Spontaneous	MF	10 mg/kg	diet	=	[76]
	C57BL/6 mice	M	Spontaneous	MF	10 mg/kg	diet	=	[76]
	LIO rats	F	Spontaneous	BF	5 mg/rat	oral	↓	[20]
	LIO rats	F	Spontaneous	PF	2 mg/rat	oral	↓	[21]
	LIO rats	F	X-rays	PF	5 mg/rat	oral	↓	[30, 36]
	F344 rats	M&F	Spontaneous	PF	400-800 ppm	diet	=	[60]

Notes: M, Male; F, Female.

1 Carcinogenic agents: AOM, azoxymethane; BP, benzo(a)pyrene; DMBA, 7,12-dimethylbenz(a)anthracene; DMH, 1,2-dimethylhydrazine; DSS, dextrane sodium sulphur; E2, estradiol-17β; HCHFD, high carbohydrate fat diet; HER-2/neu, transgen; LID, liver-IGF-1-deficient mice; MCA, 20-methylcholanthrene; MMTV, murine mamary tumor virus; NBOPA, N-nitrosobis(2-oxopropyl)amine; NDEA, N-nitrosodiethylamin; NEU, N-nitrosoethylurea; NMU, N-nitrosomethylurea; NKK, 4–(methylnitroosamino)–1–(3-pyridyl)-1-butanone; 4-NQO, 4–nitroquinoline-1-oxide; STZ, streptozotocin; TPA,12-O-tetradecanoylphorbol-13-acetate.

2 Drugs: BF. buformin; MF, metformin; PF, phenformin.

3 Route of administration: diet, with lab chow; d.w., drinking water; i.p., intraperitoneally; oral, gavage; s.c., subcutaeously; tr.pl., transplacental.

4 Effect: ↓, inhibition of tumor incidence and/or tumor multiplicity, and/or increase of tumor latency; =, no effect.

### Impact of dose and route of administration on effects of metformin

The selection of adequate dose of MF is one of most discussed questions. Some authors believe that the doses of MF used in *in vitro* studies much more than concentration of MF in the blood of patients.[15] Graham et al. [105] have shown that after a single dose a peak plasma concentration of MF in T2D patients was in ranges from 0.5 to 2 μg/mL (4 μM to 15 μM). In the majority of *in vitro* studies MF inhibited tumor growth in doses from 0.5 to 50 mM. It seems really much more than in human. In studies on antitumor effect using xenografs of human tumors into athymic nude mice as well as with transplantable tumor lines, MF was given with drinking water in the concentrations from 0.1 to 5 mg/mL, orally or intraperitoneally in doses of from 50 to 300 mg/kg of the body weight. [92, 93, 96, 97] In the studies of anticarcinogenic potential of MF it was administered with drinking water in concentrations from 0.1 to 1 mg/mL, with diet in doses from 800 to 2000 ppm, orally in doses from 100 to300 mg/kg of the body weight (Table 1). After repeated administration, MF does not accumulate in the plasma and did not bind to plasma proteins. Biodistribution of [14]C-labeled MF has been studied in mice 1 hour after intraperitoneal (i.p.) injection (250 mg/kg) or gavage (5 mg/mL), and in mice allowed to drink oral MF dissolved in water (5 mg/mL) *ad libitum* during 5 days. [63] The authors observed practically similar patterns of MF distribution after a single i.p. or gavage administration with MF levels about 4.5 times higher in liver than in lungs. Absolute levels of MF was 6.1 μM in plasma of mice that were allowed to drink MF *ad libitum* whereas in mice exposed to a single i.p. injection or gavage −29.1 and 12.27 μM respectively. It was estimated that human equivalent dose (milligram per kilogram) equal to animal dose (milligram per kilogram) x animal *K_m_*/ human *K_m_*, where species and *K_m_* values are based on body surface area (*K_m_* for adult human weighted 60 kg is 37, and *K_m_* for mouse weighted 20 g is 3.[106] Tan et al. [69] calculated that daily i.p. administration of 125 mg/kg MF into mice is equivalent to human dose 600 mg/average size person of 60 kg and concluded that the dose of 125 mg/kg is approximately 4 times less than the maximum safe dose of MF (2500 mg/day). Thus, doses MF less than 500 mg/kg in mice yielded plasma level of MF practically similar to that in diabetic patients. Our calculation have shown that used in our experiments dose of MF 100 mg/kg equal to 300 mg/m^2^ of the surface area. Recalculation for humans gives in average 510 mg/m^2^, that is much less that commonly used in clinical practice (1.0 – 2.5 g per day). We believe that studies on the effect of various routes of metformin administration at various doses levels will very helpful for a search most optimal regimens for prevention and treatment of cancer.

## CONCLUSIONS

The history of biguanides in oncology started in 70th of last century at N.N. Petrov Research Institute of Oncology by Dilman and his colleagues is rather dramatic and seems not came to “a happy end” at present time. First publications in 1974-1982 showing the high potential of PF and BF in prevention of spontaneous and induced by chemical carcinogens, X-rays and viruses carcinogenesis were not met an interest adequate to the degree of real importance of these finding. Whereas the both *in vitro* and *in vivo* experiments provide new evidences of anti-carcinogenic potential of biguanides, and the majority of clinical observations clearly demonstrates protective effect of MF in relation to many localization of cancer (see Box 1), there are some publications on results of clinical trials that are inconclusive and sometime were demonstrated adverse effect of MF. [34, 107] Recently Bio Med Central Biology published interview with N. Chandel [108] who as an explanation of possible reasons for this inconsistence cited rather sardonic comment of M. Pollack – one of the leaders in the topic: “The problem with metformin is it's cheap, it's widely available, it has a great safety profile, and anyone can use it”. Really, it is difficult to say better… In PubMed, under the words such as “metformin and cancer” the number of indexed papers were increasing exponentially from zero in 1990 to more than 2500 last September. Among them around 185 reviews on the topic were published only during last 5 years. So there are too many works and still there is no final conclusion. May be the time became to make this long story short. We believe that efficacy of MF should be evaluated according to criteria, experience and rules of the WHO International Agency for Research on Cancer.

**Box 1:** Antidiabetic biguanides as geroprotectors and anti-carcinogens: milestones

Year	Phenomenon being shown in the first time
1971	Vladimir Dilman originally developed idea that antidiabetic biguanides may be promising as geroprotectors and anticancer drugs [16]
1974	Phenformin inhibits mammary carcinogenesis induced by DMBA in rats [17]
1977	Phenformin alleviates metabolic immunodepression induced by DMH in rats [25]
1979	Phenformin inhibits spontaneous carcinogenesis and increases of the life span in female C3H/Sn mice [18]
1980	Buformin inhibits spontaneous carcinogenesis, postpones of estrus cycle swithching-off and increases the life span in female rats [20]
1980	Buformin inhibits transplacental carcinogenesis induced by NMU in rats [27]
1982	Phenformin inhibits spontaneous carcinogenesis and the increase of the life span in female rats [21]
1982	Phenformin inhibits carcinogenesis induced by X-rays irradiation in rats [30]
2001	Metformin inhibits pancreatic carcinogenesis induced by NBOPA in hamsters [35]
2005	Metformin inhibits spontaneous carcinogenesis and the increase of the life span in female HER-2/neu transgenic mice [37]
	Metformin decreases the risk of cancer in type 2 diabetes patients [38, 39]
2008	Metformin increases life span in outbred female SHR [49]
2008-2014	Treatment with metformin prevent spontaneous and/or induced carcinogenesis in: 2008: small intestines [79]; lymphoid tissue [79] 2009: uterus [58] 2010: cervix [61]; skin [54]; soft tissues [57]; lung [62]; pancreatic islets [70]; colon [80] 2012: oral mucosa [66]; liver [71] 2013: pancreas [67] 2014: kidney [65]
2014	Diabetes type 2 patients treated with metformin monotherapy have 15% longer survival than matched controls without diabetes [5]
2015	Announcing the project TAME (Targeting Aging with Metformin) suggesting delay age-related diseases and increase survival in elderly people [3, 4]
